# Supplementary figures and images for: Nucleo-cytoplasmic shuttling of splicing factor SRSF1 is required for development and cilia function
Source: eLife. 2021 Aug 2;10:e65104. doi: 10.7554/eLife.65104 (PMC8352595; doi:10.7554/eLife.65104)

WB: SRSF1

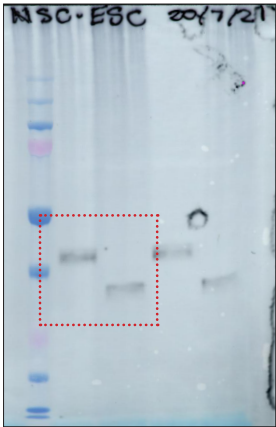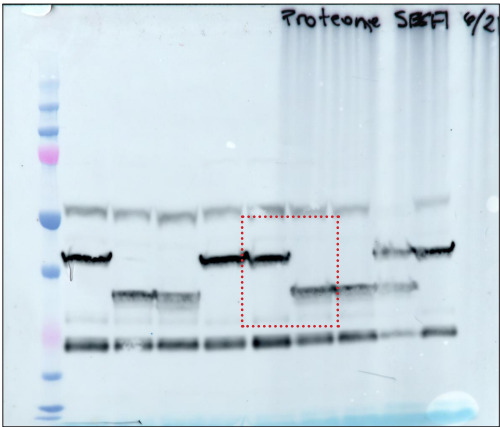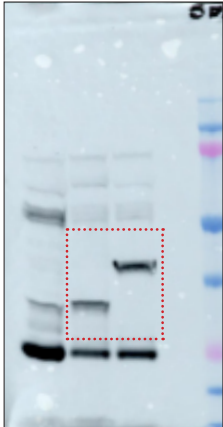

WB:  $\alpha$  Tubulin

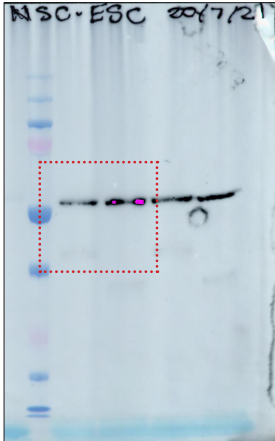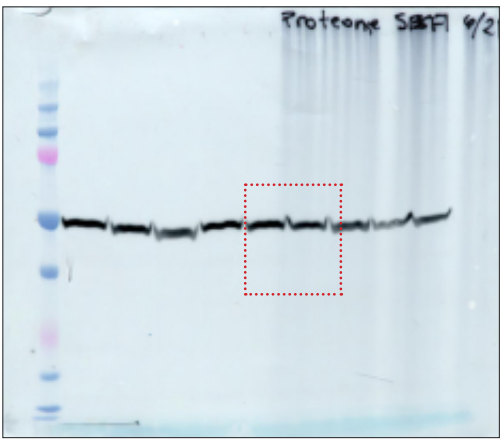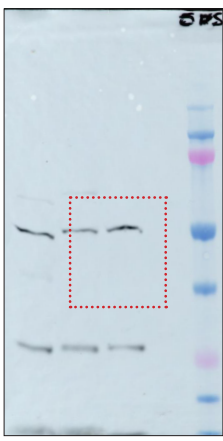

NSCs

Testes (P22)  
used in proteome analysis

Trachea  
(P23)

Supplement: Figure 1—source data 1. [file elife-65104-fig1-data1.pdf]
