# Supplementary material for: Nucleo-cytoplasmic shuttling of splicing factor SRSF1 is required for development and cilia function
Source: eLife. 2021 Aug 2;10:e65104. doi: 10.7554/eLife.65104 (PMC8352595; doi:10.7554/eLife.65104)
Supplement: Figure 1—figure supplement 1—source data 1. [file elife-65104-fig1-figsupp1-data1.pdf]

SRSF1 antibody

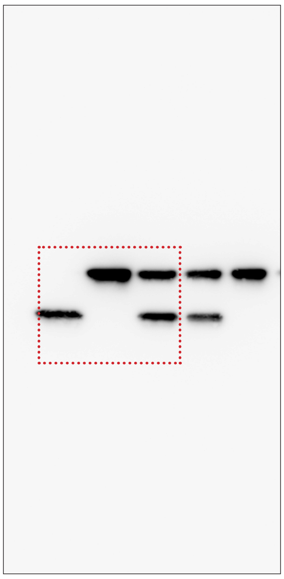

WB: SRSF1

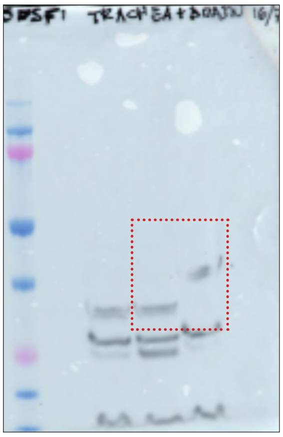

Brain
(P23)

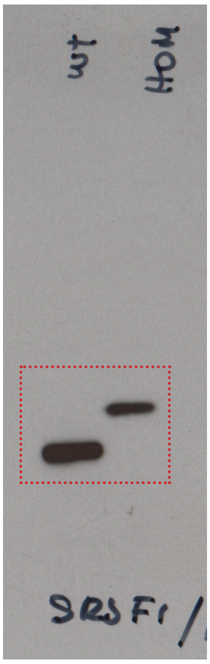

WB: SRSF1

T7 antibody

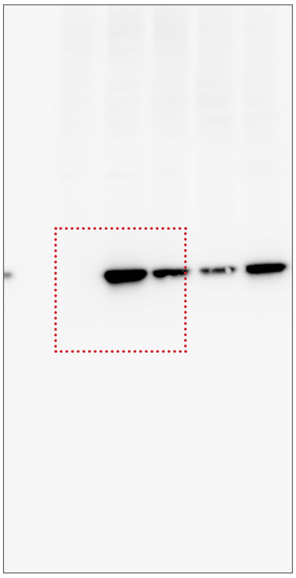

WB: T7

ESCs

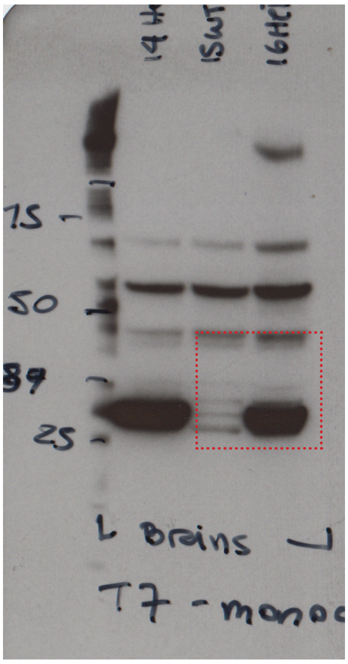

WB: T7

Brains

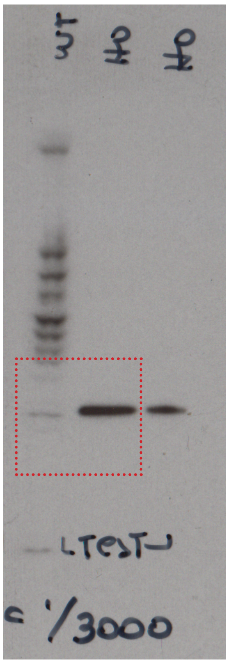

WB: T7

Testes

Brain and Testes samples developed with T7 are from different animals than those developed with SRSF1 antibody.
