# Supplementary material for: Nucleo-cytoplasmic shuttling of splicing factor SRSF1 is required for development and cilia function
Source: eLife. 2021 Aug 2;10:e65104. doi: 10.7554/eLife.65104 (PMC8352595; doi:10.7554/eLife.65104)
Supplement: Transparent reporting form [file elife-65104-transrepform.pdf]

eLIFE
1st Floor
24 Hills Road
Cambridge CB2 1JP, UK
P 01223 855340
W elifesciences.org
T @elife

eLife's transparent reporting form

We encourage authors to provide detailed information *within their submission* to facilitate the interpretation and replication of experiments. Authors can upload supporting documentation to indicate the use of appropriate reporting guidelines for health-related research (see EQUATOR Network), life science research (see the BioSharing Information Resource), or the ARRIVE guidelines for reporting work involving animal research. Where applicable, authors should refer to any relevant reporting standards documents in this form.

If you have any questions, please consult our Journal Policies and/or contact us: editorial@elifesciences.org.

Sample-size estimation

- You should state whether an appropriate sample size was computed when the study was being designed
- You should state the statistical method of sample size computation and any required assumptions
- If no explicit power analysis was used, you should describe how you decided what sample (replicate) size (number) to use

Please outline where this information can be found within the submission (e.g., sections or figure legends), or explain why this information doesn't apply to your submission:

There was no prior expectation of effect of sample size, power analysis was not used either. In general, three replicates were used due to practical limitations; for example Tracheal primary cultures have a finite number of cell divisions where control and mutants must be grown in parallel. A compromise is needed between number of time points/animal and number of replicates for proteomics, live imaging and immunofluorescence. Likewise, for collection of P22-23 testes, numbers of males available were compromised due to premature death or early termination based on health concerns, the remaining males have to be used either for polysome shift analysis or total proteomes. In cases when more replicates have been used, this is stated in the relevant text.

Replicates

- You should report how often each experiment was performed
- You should include a definition of biological versus technical replication
- The data obtained should be provided and sufficient information should be provided to indicate the number of independent biological and/or technical replicates
- If you encountered any outliers, you should describe how these were handled
- Criteria for exclusion/inclusion of data should be clearly stated
- High-throughput sequence data should be uploaded before submission, with a private link for reviewers provided (these are available from both GEO and ArrayExpress)

Please outline where this information can be found within the submission (e.g., sections or figure legends), or explain why this information doesn't apply to your submission:

eLife Sciences Publications, Ltd is a limited liability non-profit non-stock corporation incorporated in the State of Delaware, USA, with company number 5030732, and is registered in the UK with company number FC030576 and branch number BR015634 at the address 1st Floor, 24 Hills Road, Cambridge CB2 1JP | August 2014

Information on number of replicates can be found in Results and Material & Methods sections and also in Figure legends. Information related to deposited data is provided in the Key resources table. All experiments done with animal tissues are described as number of animals used for each genotypes for each experiment and time point. 3-5 independent replicates (i.e. 'biological' replicate) were used, depending on the experiment. An independent experiment is defined as an experiment carried out on a different day, using freshly prepared biological samples.

Statistical reporting

- Statistical analysis methods should be described and justified
- Raw data should be presented in figures whenever informative to do so (typically when N per group is less than 10)
- For each experiment, you should identify the statistical tests used, exact values of N, definitions of center, methods of multiple test correction, and dispersion and precision measures (e.g., mean, median, SD, SEM, confidence intervals; and, for the major substantive results, a measure of effect size (e.g., Pearson's r, Cohen's d)
- Report exact p-values wherever possible alongside the summary statistics and 95% confidence intervals. These should be reported for all key questions and not only when the p-value is less than 0.05.

Please outline where this information can be found within the submission (e.g., sections or figure legends), or explain why this information doesn't apply to your submission:

Information on statistical analysis can be found in Results and Material & Methods sections and Figure legends. General information on data analysis is given at end of the Methods section. Every figure legend has statistical information concerning data representation, details of statistical tests used and exact p-values. In some cases, Prism (the software used for statistical analysis) does not give an exact number, this is presented as shown by the software.

We have uploaded raw data to repositories as well as an Excel table with the values plotted in each of the relevant panels.

(For large datasets, or papers with a very large number of statistical tests, you may upload a single table file with tests, Ns, etc., with reference to sections in the manuscript.)

Group allocation

- Indicate how samples were allocated into experimental groups (in the case of clinical studies, please specify allocation to treatment method); if randomization was used, please also state if restricted randomization was applied
- Indicate if masking was used during group allocation, data collection and/or data analysis

Please outline where this information can be found within the submission (e.g., sections or figure legends), or explain why this information doesn't apply to your submission:

No randomization was used. Groups were allocated based on genotyping.
For data analysis, particularly counts and acquisition of images, random fields of view were chosen and scoring was done post-acquisition, this is described in methods.

eLife Sciences Publications, Ltd is a limited liability non-profit non-stock corporation incorporated in the State of Delaware, USA, with company number 5030732, and is registered in the UK with company number FC030576 and branch number BR015634 at the address 1st Floor, 24 Hills Road, Cambridge CB2 1JP | August 2014

eLIFE
1st Floor
24 Hills Road
Cambridge CB2 1JP, UK
P 01223 855340
W elifesciences.org
T @elife

Additional data files ("source data")

- We encourage you to upload relevant additional data files, such as numerical data that are represented as a graph in a figure, or as a summary table
- Where provided, these should be in the most useful format, and they can be uploaded as "Source data" files linked to a main figure or table
- Include model definition files including the full list of parameters used
- Include code used for data analysis (e.g., R, MatLab)
- Avoid stating that data files are "available upon request"

Please indicate the figures or tables for which source data files have been provided:

Source data files have been provided for figures 1, 4 and 5, and Figure 1-figure supplement 1 and Figure 4-figure supplement 1

eLife Sciences Publications, Ltd is a limited liability non-profit non-stock corporation incorporated in the State of Delaware, USA, with company number 5030732, and is registered in the UK with company number FC030576 and branch number BR015634 at the address 1st Floor, 24 Hills Road, Cambridge CB2 1JP | August 2014
